# Operational parameters optimization for remediation of crude oil-polluted water in floating treatment wetlands using response surface methodology

**DOI:** 10.1038/s41598-022-08517-1

**Published:** 2022-03-16

**Authors:** Khadeeja Rehman, Muhammad Arslan, Jochen A. Müller, Muhammad Saeed, Samina Anwar, Ejazul Islam, Asma Imran, Imran Amin, Tanveer Mustafa, Samina Iqbal, Muhammad Afzal

**Affiliations:** 1 Soil & Environmental Biotechnology Division, National Institute for Biotechnology and Genetic Engineering College, Pakistan Institute of Engineering and Applied Sciences (NIBGE-C, PIEAS), Faisalabad, Punjab 38000 Pakistan; 2 Pakistan Institute of Engineering and Applied Sciences (PIEAS), Islamabad, Pakistan; 3 Civil and Environmental Engineering Department, University of Alberta, Edmonton, AB T6G 2W2 Canada; 4 Institute for Biological Interfaces (IBG 5), Karlsruhe Institute of Technology, Hermann von Helmholtz Platz 1, 76344 Eggenstein-Leopoldshafen, Germany; 5 Agricultural Biotechnology Division, National Institute for Biotechnology and Genetic Engineering College, Pakistan Institute of Engineering and Applied Sciences (NIBGE-C, PIEAS), Faisalabad, Punjab 38000 Pakistan

**Keywords:** Plant sciences, Biotechnology, Environmental biotechnology

## Abstract

The application of floating treatment wetlands (FTWs) is an innovative nature-based solution for the remediation of polluted water. The rational improvement of water treatment via FTWs is typically based on multifactorial experiments which are labor-intensive and time-consuming. Here, we used the response surface methodology (RSM) for the optimization of FTW’s operational parameters for the remediation of water polluted by crude oil. The central composite design (CCD) of RSM was used to generate the experimental layout for testing the effect of the variables hydrocarbon, nutrient, and surfactant concentrations, aeration, and retention time on the hydrocarbon removal in 50 different FTW test systems planted with the common reed, *Phragmites australis*. The results from these FTW were used to formulate a mathematical model in which the computational data strongly correlated with the experimental results. The operational parameters were further optimized via modeling prediction plus experimental validation in test FTW systems. In the FTW with optimized parameters, there was a 95% attenuation of the hydrocarbon concentration, which was very close to the 98% attenuation predicted by the model. The cost-effectiveness ratio showed a reduction of the treatment cost up to $0.048/liter of wastewater. The approach showed that RSM is a useful strategy for designing FTW experiments and optimizing operational parameters.

## Introduction

The petroleum industry produces approximately 5.3 million m^3^ of oil-contaminated water per day during drilling, extraction, and refining processes^1,2^. This volume of wastewater will remain high in the near future since the combustion of petroleum, although unsustainable, accounts for about one-third of the energy budget of the world’s societies^3^. At many oil exploration sites, wastewater enriched with crude oil is stored in evaporation pits and eventually discharged into the environment without further treatment^4^. The (eco)toxicological effects of many crude oil constituents render the remediation of such contaminated water a pressing need in oil-producing countries^5,6^. Conventional remediation methods based on physical and chemical processes come with substantial demands for energy input, capital investments, as well as operational and maintenance costs, and are therefore rarely used^4,7–10^.

Nature-based solutions (NBS) are viable alternatives to conventional approaches for the remediation of oil-contaminated water^4,11^. For example, NBS have been successfully applied in Oman, where 3.6 million m^3^ of treatment wetlands are used for the remediation of wastewater polluted with crude oil^12^. Many studies suggest that Floating Treatment Wetlands (FTW) are a highly effective NBS for the remediation of polluted water, including hydrocarbon-enriched wastewater^4,6,13^. The treatment success of FTW relies on synergistic interactions between plants, growing as buoyant mats, and their associated microbial communities^4,13,14^. In this partnership, plants provide the microbial communities with nutrients, oxygen, and residency for their improved survival and catabolic activities in the rhizo- and endosphere^15,16^. In turn, microorganisms transform toxic compounds including hydrocarbons into innocuous compounds and may have various plant growth-promoting capabilities^17^.

High concentrations of hydrocarbons in the water endanger the health of plants and associated bacteria and therefore reduce the remediation efficiency of FTW^11,18,19^. There are several possible remedies for the reduced treatment efficiency, some of which have already been tested with hydrocarbon-contaminated water and also with soil systems. There was a positive effect on hydrocarbon transformation through the addition of nutrients, surfactant amendments, aeration, and increased hydraulic retention time^11,20–23^. Typically, the concentrations of nutrients, and surfactants, as well as the adjustment of operational parameters such as retention time are selected only based on theoretical knowledge and heuristics. It is conceivable that hydrocarbon degradation in FTW can be increased cost-efficiently by optimizing the extent of the different improvement measures. However, empirical identification of the optimized parameters would require that several combinations of variables are tested. Such a multifactorial experiment is often not feasible. Therefore, there is a need for an efficient experimental design.

Response Surface Methodology (RSM) is a collection of statistical techniques for designing experiments and consists of different stages such as evaluating the effects of variables and finding optimum conditions via generating response surfaces and contour plots^24^. RSM helps to examine the interactive effects between variables and to build a mathematical model that can represent the entire process under study^25^. The central composite design (CCD) is the most commonly used fractional factorial design used in the response surface model. In this design, the central points are augmented with a group of axial points, also known as star points. With this design, first-order and second-order terms can be estimated quickly^26–29^. Previously, several wastewater remediation processes have been optimized with RSM for maximum removal of organic and inorganic pollutants from wastewater^30–32^. However, optimization of water treatment with FTWs using RSM has not been carried out.

In this study, for the first time, we used CCD of RSM to optimize the operational parameters in FTW for maximum remediation at reduced costs. To this end, we generated the experimental layout for multi-factorial tests of hydrocarbon degradation in FTW, then carried out tests at mesocosm scale, modeled experimental data with RSM, and validated the modeling prediction at the mesocosm scale. The experimental results fitted well with the model prediction, showcasing that RSM is a useful tool that can help to select FTW’s operational parameters for the optimized remediation of hydrocarbon-contaminated water. Finally, the cost-effectiveness ratio (CER) was calculated to support the usefulness of RSM in terms of parameters optimization for a full-scale experiment^33^.

## Results and discussion

### RSM experimental design and hydrocarbon degradation in planted mesocosms

First, the test values of the five variables nutrients (A), surfactant (B), aeration (C), hydrocarbon content (D), and hydraulic retention time (E) were chosen at three levels [low (− 1), central (0), and high (+ 1)] based on previous studies (Table 1)^11,20–23^. Then, CCD was used to generate the experimental design matrix. CCD was favored over a Box Behnken design (BBD) because it offers more axial design points compared to the BBD while being suitable for testing five variables^34^. Furthermore, CCD is better at extreme conditions and gives better results for quadratic models^35^. In this study, the matrix consisted of 32 factorial points, 10 axial points, and 8 central points, resulting in a total of 50 experimental setups (Table 2).

**Table 1:** Summary of experimental factors and design.

Variable	Unit	Level		
		−1	0	+ 1
Nutrients, A	Ratio	0	1	2
Surfactant, B	%	0	0.005	0.01
Aeration, C	L/min	0	1	2
Hydrocarbons content, D	%	0.5	0.75	1
Retention time, E	Days	8	16	24

**Table 2:** Central composite design matrix for the five independent variables with the observed and predicted response for oil removal, COD reduction and plant biomass.

Run	HCs	Sur	Aer	Nut	RT	HCs reduction		COD reduction		Plant biomass	
	mg L^−1^	%	Liter min^−1^	Ratio	Days	EV (%)	PV (%)	EV (%)	PV (%)	EV (g)	PV (g)
1	1	0.01	2	2	8	28	28.20	37.00	41.21	23.00	22.87
2	0.75	0.005	1	0	16	57	46.38	67.00	58.58	28.00	26.49
3	1	0.01	2	0	24	66	72.08	77.00	76.25	29.00	30.79
4	0.5	0.01	0	0	24	64	60.84	76.00	69.54	31.00	29.45
5	0.75	0.005	2	1	16	61	59.60	71.00	71.28	29.00	28.67
6	0.5	0	0	2	24	61	53.88	72.00	67.20	30.00	27.62
7	0.5	0.01	2	2	8	37	32.72	49.00	46.80	24.00	25.11
8	0.5	0.01	0	0	8	24	22.47	33.00	36.14	22.00	24.26
9	1	0.01	0	2	8	21	26.21	30.00	28.92	23.00	22.80
10	0.75	0.005	1	2	16	53	58.37	59.00	67.69	27.00	29.02
11	0.5	0	2	0	24	59	54.61	67.00	59.63	29.00	26.92
12	1	0	2	2	8	21	23.04	29.00	30.13	23.00	20.51
13	0.5	0	0	2	8	17	18.32	23.00	23.42	20.00	22.68
14	0.75	0.005	1	1	16	77	69.72	88.00	78.62	36.00	34.75
15	0.75	0.005	1	1	16	77	69.72	85.00	78.62	36.00	34.75
16	0.75	0.005	1	1	16	73	69.72	80.00	78.62	35.00	34.75
17	1	0.01	2	0	8	25	29.48	36.00	42.85	21.00	22.09
18	0.5	0	0	0	24	19	30.80	31.00	47.33	21.00	23.34
19	0.5	0	2	2	24	71	68.23	83.00	79.49	33.00	31.20
20	0.75	0.005	1	1	16	71	69.72	81.00	78.62	35.00	34.75
21	1	0	0	2	8	16	14.98	24.00	17.84	19.00	20.44
22	0.75	0.005	0	1	16	49	45.02	59.00	58.99	26.00	26.85
23	0.5	0.01	2	0	24	73	79.21	81.00	81.84	34.00	33.03
24	0.75	0.005	1	1	8	43	46.51	57.00	59.33	46.00	39.85
25	0.5	0	2	2	8	23	27.14	33.00	35.72	22.00	22.75
26	1	0	0	2	24	57	48.02	64.00	61.61	27.00	25.39
27	1	0	0	0	8	9	4.33	16.00	8.34	21.00	19.66
28	0.5	0.01	0	2	8	29	30.58	38.00	34.51	26.00	25.04
29	0.75	0.005	1	1	16	75	69.72	82.00	78.62	36.00	34.75
30	1	0	2	0	8	16	15.37	26.00	20.64	21.00	19.73
31	0.5	0.01	2	0	8	34	34.11	45.00	48.43	23.00	24.33
32	1	0	0	0	24	20	26.32	29.00	41.74	18.00	21.11
33	0.5	0.005	1	1	16	67	73.27	70.00	81.42	32.00	32.38
34	0.75	0.005	1	1	16	79	69.72	81.00	78.62	37.00	34.75
35	1	0.01	0	2	24	61	66.91	72.00	72.70	30.00	31.50
36	1	0	2	2	24	59	61.62	68.00	73.91	29.00	28.96
37	1	0.01	0	0	8	19	18.75	29.00	30.55	22.00	22.02
38	0.5	0	0	0	8	6	6.20	11.00	13.93	23.00	21.90
39	0.75	0.005	1	1	16	69	69.72	83.00	78.62	34.00	34.75
40	0.5	0	2	0	8	19	18.75	27.00	26.22	19.00	21.97
41	1	0.01	2	2	24	73	69.89	81.00	84.99	32.00	35.07
42	1	0	2	0	24	55	48.72	63.00	54.04	27.00	24.68
43	1	0.01	0	0	24	57	54.61	66.00	63.95	30.00	27.22
44	0.5	0.01	0	2	24	76	73.79	82.00	78.28	36.00	33.73
45	0.75	0.005	1	1	16	76	69.72	86.00	78.62	36.00	34.75
**46**	**0.75**	**0.005**	**1**	**1**	**24**	**89**	**97.61**	**96.00**	**97.92**	**40.00**	**46.67**
47	0.75	0	1	1	16	43	58.68	58.00	70.30	27.00	32.63
48	1	0.005	1	1	16	61	66.26	70.00	75.83	30.00	30.14
49	0.75	0.01	1	1	16	79	81.72	84.00	86.95	37.00	36.86
50	0.5	0.01	2	2	24	83	76.91	91.00	90.58	38.00	37.31

*EV* experimental value, *PV* predicted value, *HCs* hydrocarbons, *Sur* surfactant, *Aer* aeration, *Nut* nutrients, *RT* retention time, *Fv* f-value, *pv* p-value.

Significance values are given in bold.

**Figure 1:**
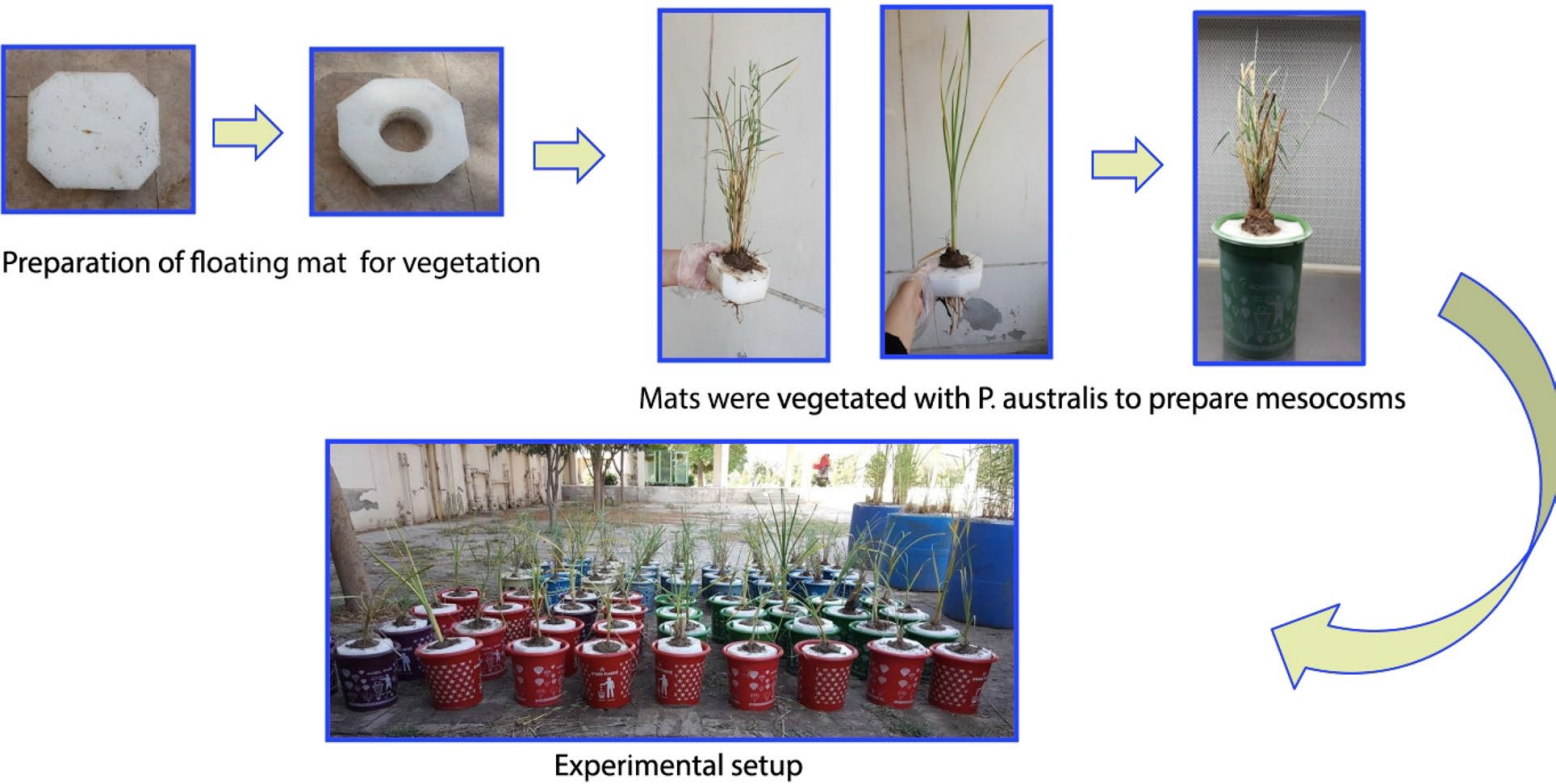
Preparation of mesocosms with *P. australis* for the optimization of FTW operational parameters.

The 50 different setups were established in triplicates as 3-L mesocosms with hydroponically grown common reed (*Phragmites australis*) (Fig. 1). Table 2 shows the results of hydrocarbon removal (% concentration reduction), COD reduction (%), and growth of plant biomass (g) in the setups. The highest hydrocarbon removal (89%) occurred with A: 14 mg L^−1^ nitrogen and 1.9 mg L^−1^ phosphorus; B: 0.005% (w/v) of sodium dodecyl sulphate as surfactant; C: 1 L of air min^−1^; D: 0.75% hydrocarbon content; and E: 24 days (setup #46). The lowest hydrocarbon removal among all 50 setups was 6% (setup # 38 = HC: 0.5 mg/L, surfactant: 0%, aeration: 0 L/min, nutrients ratio: 0, and retention time: 8 days), and the lowest removal with a hydraulic retention time of 24 days was 19% (setup # 18 = HC: 0.5 mg/L, surfactant: 0%, aeration: 2 L/min, and nutrients ratio: 2), which was a substantial difference among these two setups.

### Development and validation of response surface models

Next, RSM was applied for the mathematical model building of the experimental data obtained with the hydroponic systems. Multiple regression analysis was used to test for linear (A, B, C, D, E), quadratic (A^2^, B^2^, C^2^, D^2^, E^2^) and interactive (AB, AC, AD, AE, BC, BD, BE, CD, CE, DE) effects of all variables. The following polynomial quadratic equations fitted best to the experimental response data for hydrocarbon degradation, COD reduction, and increase in plant biomass (Eqs. 1–3).

1 $${\text{Y}}_{{{\text{hydrocarbon reduction (\% )}}}} = \, + { 8}.{35 }{-} \, 0.{2}0{\text{5 A }} + \, 0.{\text{689 B }} + \, 0.{5}0{\text{6 C }} + \, 0.{\text{417 D }} + { 1}.{\text{53 E }}{-} \, 0.{\text{186 BC}}{-}0.{25}0{\text{ BD }}{-} \, 0.{\text{229 CD }}{-}{ 1}.{\text{13 C}}^{{2}} {-}{ 1}.{\text{12 D}}^{{2}} .$$

2 $${\text{Y}}_{{{\text{COD reduction }}(\% )}} = \, + { 78}.{62 }{-}{ 2}.{\text{79 A }} + { 8}.{\text{32 B }} + { 6}.{\text{15 C }} + { 4}.{\text{56 D }} + {19}.{\text{29 E }}{-}{ 2}.{\text{78 BD }} + { 2}.{\text{59 DE }}{-}{ 13}.{\text{49 C}}^{{2}} {-}{ 15}.{\text{49 D}}^{{2}} .$$

3 $${\text{Y}}_{{{\text{plant biomass }}({\text{g}})}} = \, + { 34}.{75 }{-}{ 1}.{\text{12 A }} + { 2}.{\text{12 B }} + \, 0.{\text{9118 C }} + { 1}.{\text{26 D }} + { 3}.{\text{41 E }} + \, 0.{\text{937 BE }} + \, 0.{\text{875 CE }} + \, 0.{\text{875 DE }}{-}{ 3}.{\text{49 A}}^{{2}} {-}{ 6}.{\text{99 C}}^{{2}} {-}{ 6}.{\text{99 D}}^{{2}} + { 8}.{\text{51 E}}^{{2}} .$$

where Y is the response value, A stands for nutrient concentration, B for surfactant concentration, C for aeration, D for hydrocarbon content, and E for retention time; AB, AC, AD, AE, BC, BD, BE, CD, CE, and DE are the interaction effects; A^2^, B^2^, C^2^, D^2^, and E^2^ represent square effects. The negative (−) and positive (+) signs of regression coefficients showed that there were antagonistic and synergistic effects of the variables. Insignificant terms with p > 0.05 were removed from the three models.

All five variables possessed the same linear significant terms A, B, C, D, and E and quadratic terms C^2^ and D^2^. The interaction terms BC, BD, and CD were significant for hydrocarbon reduction, BD and DE were significant for COD reduction and BE was the only significant interaction term for the production of plant biomass.

**Figure 2:**
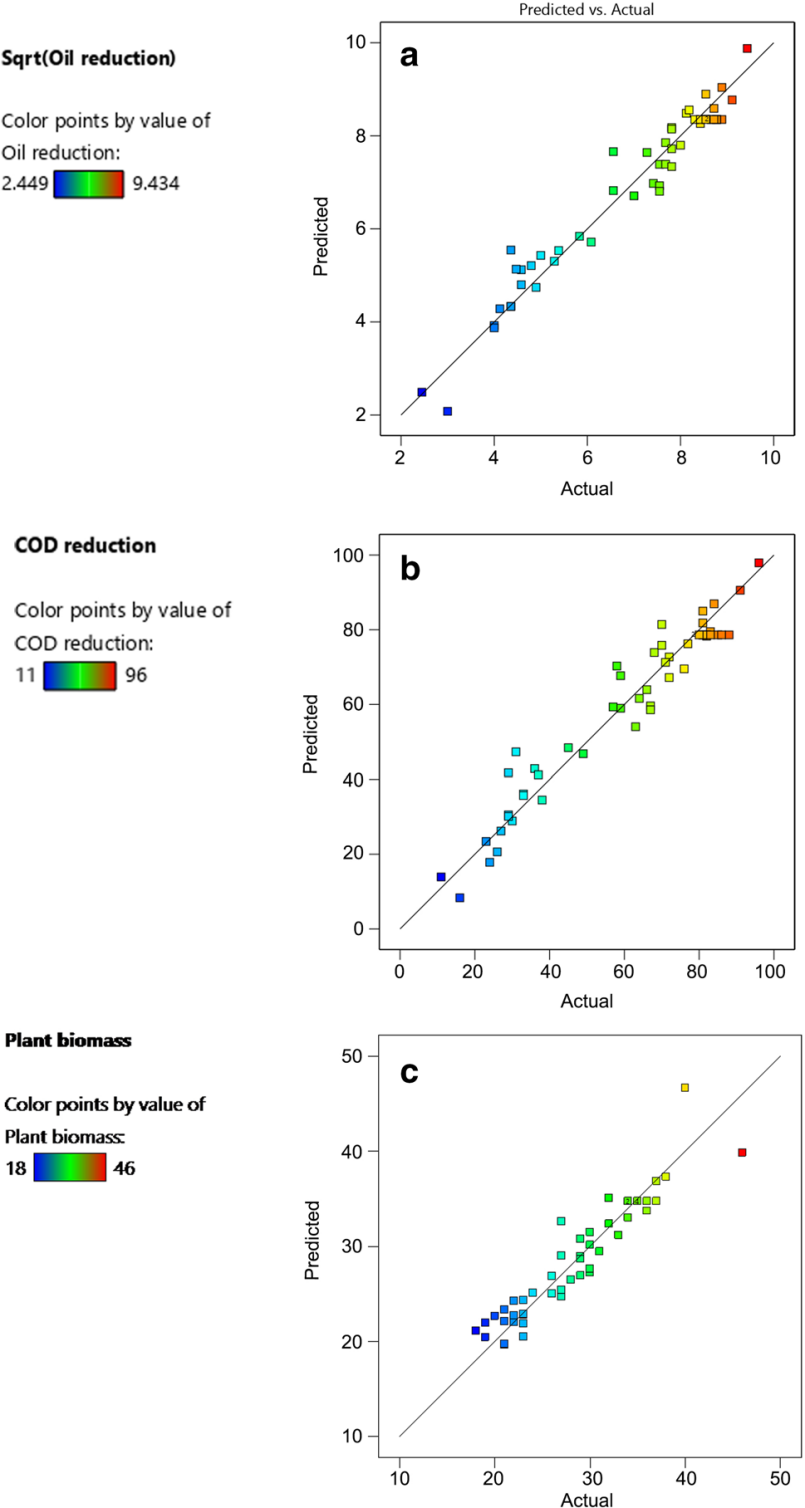
(**a–c**) Correlation between the actual and predicted (**a**) hydrocarbon reduction, (**b**) COD reduction, and (**c**) increase in plant biomass.

An analysis of variance (ANOVA) confirmed the adequacy of the quadratic models for the three responses with p-values < 0.0001 (Table 3). Precisely, hydrocarbons attenuation, COD reduction, and gain in plant biomass were tested by fitting quadratic models in RSM. This approach describes the mathematical relationship between each term in the model and response. The coefficient of determination (*R*^2^) was 0.95 for the attenuation of the hydrocarbon concentration (Fig. 2a). For COD reduction and for increase in plant biomass it was *R*^2^ = 0.93 and *R*^2^ = 0.88, respectively (Fig. 2b,c). The independent variables accounted for 96% of the variability. Furthermore, there were strong relations of surfactant, aeration, and nutrients with *R*^2^ values of 0.95, 0.939, and 0.883, respectively (Table 2). The goodness-of-fit of the regression equation was confirmed by the high value of the adjusted determination coefficient (*R*^2^_adj_ = 0.938). This high value showed that the selected factors and their values constitute a very good representation of the main processes that influence the hydrocarbon treatment efficiency of the FTW systems.

**Table 3:** ANOVA of the quadratic model for hydrocarbons and COD reduction, and plant biomass production.

Source	Hydrocarbons reduction					COD reduction						Plant biomass					
	SS	df	Fv	pv		Source	SS	df	Fv	pv		Source	SS	df	Fv	pv	
Model	166.79	10	75.24	< 0.0001	Significant	Model	26,031.27	9	69.10	< 0.0001	Significant	Model	1826.14	12	23.36	< 0.0001	Significant
A-HCs	1.44	1	6.48	0.0150		A-HCs	265.44	1	6.34	0.0159		A-HCs	42.47	1	6.52	0.0149	
B-Sur	16.18	1	73.00	< 0.0001		B-Sur	2355.56	1	56.27	< 0.0001		B-Sur	152.47	1	23.40	< 0.0001	
C-Aer	8.71	1	39.31	< 0.0001		C-Aer	1284.74	1	30.69	< 0.0001		C-Aer	28.26	1	4.34	0.0442	
D-Nut	5.91	1	26.68	< 0.0001		D-Nut	706.62	1	16.88	0.0002		D-Nut	54.38	1	8.35	0.0064	
E-RT	79.32	1	357.85	< 0.0001		E-RT	12,656.94	1	302.36	< 0.0001		E-RT	395.76	1	60.74	< 0.0001	
BC	1.11	1	5.00	0.0312		BD	247.53	1	5.91	0.0196		BE	28.13	1	4.32	0.0447	
BD	2.01	1	9.06	0.0046		DE	215.28	1	5.14	0.0288		CE	24.50	1	3.76	0.0601	
CD	1.68	1	7.58	0.0089		C^2^	660.81	1	15.79	0.0003		DE	24.50	1	3.76	0.0601	
C^2^	4.68	1	21.09	< 0.0001		D^2^	871.31	1	20.81	< 0.0001		A^2^	31.86	1	4.89	0.0333	
D^2^	4.59	1	20.73	< 0.0001		Residual	1674.41	40				C^2^	127.92	1	19.63	< 0.0001	

*HCs* hydrocarbons, *Sur* surfactant, *Aer* aeration, *Nut* nutrients, *RT* retention time, *SS* sum of squares *Fv* f-value, *pv* p-value.

*R*-Squared = 0.9505; Adj *R*-Squared 0.9381; *R*-Squared = 0.9396; Adj *R*-Squared 0.9260; *R*-Squared = 0.8834; Adj *R*-Squared 0.8456; Pred *R*-Squared = 0.9169, Pred *R*-Squared = 0.9045, Pred *R*-Squared 0.7358.

### Model analysis via 2D contour graphs and 3-D surface plots

**Figure 3:**
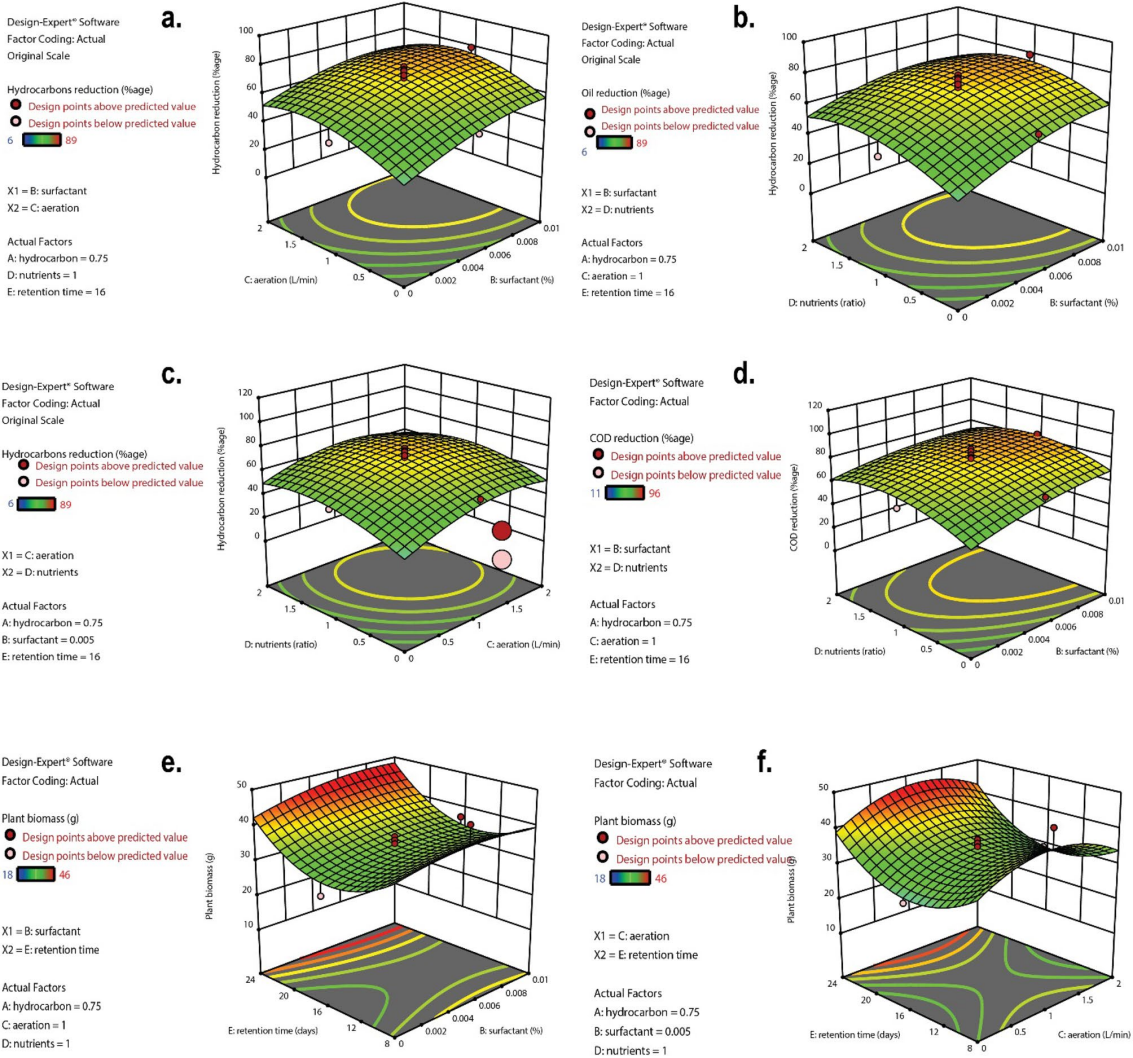
Response surface graphs for hydrocarbon reduction, COD reduction, and increase in plant biomass.

To visualize the relationships between the experimental variables and the corresponding responses, we used RSM to draw three-dimensional response surface graphs and contour plots as their two-dimensional projections (Fig. 3). In this approach, the significance of mutual effects of the experimental variables is represented by the curvature of the response surface and contour lines. Saddle and ridge-shaped 3-D graphs [(inverse) hyperbolic contour plots] exhibit a significant mutual interaction of experimental variables, while a dome-shaped 3-D graph (circular contour plot) represents a non-significant interaction. Here, the effect of the experimental factors was investigated by varying two factors over the experimental range while keeping the other three variables constant.

Figure 3 illustrates the effect of the variables on hydrocarbon decrease. The surfactant concentration and aeration significantly affected hydrocarbon reduction by varying levels of both variables. The 3-D diagram displays that hydrocarbon attenuation increases with increasing surfactant concentration whereas an increase in the level of aeration helps to decrease hydrocarbon concentration up to an optimum point (Fig. 3a). Similar results were found for the interaction between surfactant and nutrients (Fig. 3b). The ridge shape of the 3-D graph is showing a significant interaction of the variables. Higher surfactant concentrations produced a positive effect on the degradation of hydrocarbons while higher nutrient concentrations resulted in an increase in hydrocarbon attenuation to an optimum point, after which further nutrient increase caused a negative effect on the response in the model. In Fig. 3c the dome surface of the response plot shows that the interaction between nutrients and aeration is non-significant. The 3-D surface plot indicates that both high and low levels of nutrients and aeration did not have a statistically significant effect on hydrocarbon degradation.

The interactive effect of the experimental variable on COD reduction was also determined using 3D plots of RSM. The 3-D graph shows that the interaction between the variables is significant. Higher levels of surfactant had a positive effect to decrease COD in the water whereas after an optimum level a further increase in nutrients has a negative effect on the process (Fig. 3d). As expected, COD was most effectively reduced at the highest level of retention time, as shown in Fig. 3d. The effect of the variables on the growth of plant biomass was also demonstrated by the design expert. The 3-D graphs in Fig. 3e,f show that an increase in retention time increases the plant biomass significantly, while the various levels of surfactant and aeration have static or limited effects on plant biomass. A similar trend was observed for retention time, nutrients, and aeration (data not shown).

### Optimization of experimental conditions for hydrocarbon degradation

**Figure 4:**
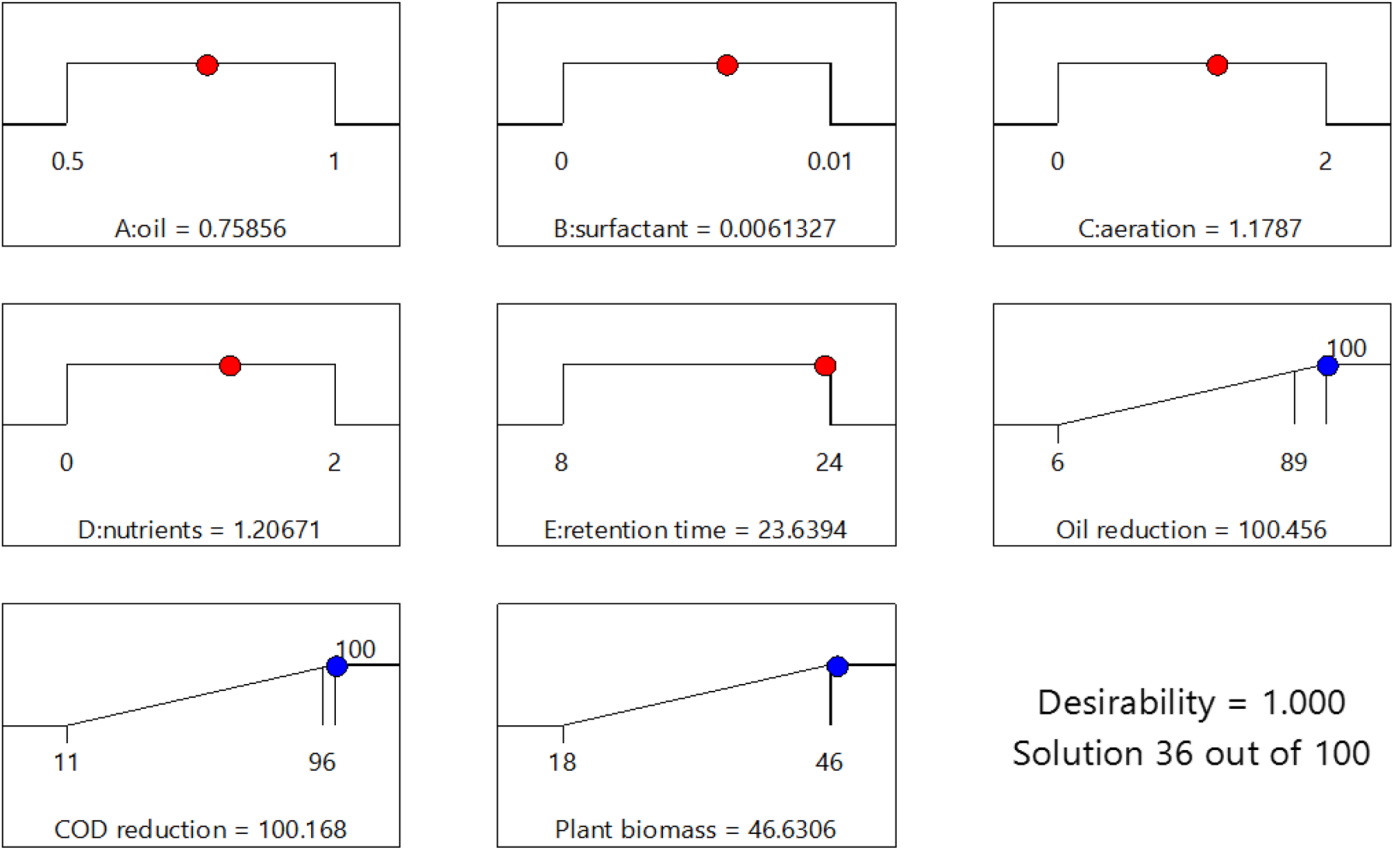
Desirability ramps for numerical optimization of hydrocarbon reduction, COD reduction and plant biomass.

Then, RSM was used to predict the optimal values of the variables namely nutrients, surfactant, aeration, hydrocarbon content with a hydraulic retention time of 24 days to maximal attenuation of the hydrocarbon concentration. The optimized values of variables predicted by the desirability function method of RSM were found to be a hydrocarbon content of 0.758%, a surfactant concentration of 0.006%, aeration of 1.178 L of air min^−1^, and a nutrient ratio of 1.20, resulting in a predicted value of hydrocarbon degradation of 98% (Fig. 4). Then we carried out another experimental test at the 3-L scale with the optimized values predicted by RSM. Attenuation of the hydrocarbon concentration of 95% was achieved in the FTW setup with the RSM-optimized operational parameters. Thus, the experimentally observed response values agree again very well with the theoretical values assumed by the model, showing the precision and accuracy of the RSM approach.

### The benefit of RSM for improved hydrocarbon degradation in FTW

In general, an RSM model can be used to predict what will happen under different conditions, but it cannot explain the mechanism of the process^34^. Nevertheless, the goodness of fit between the predicted and experimental values can indicate whether all important parameters have been accounted for in the model, and thus whether the underlying conceptual process framework is close to reality. As reported above, the adjusted determination coefficient for the model equations in this study were *R*^2^_adj_ = 0.938 with probability values of p < 10^–4^, demonstrating the significance of the model to predict the responses and thus fostering a rational and cost-effective improvement of FTW-based water treatment of oil-contaminated water at field scale. It is important that the two operational parameters *retention time* and *level of aeration* could be successfully modeled with RSM, as these are prime parameters in process engineering. These parameters of the system can be more readily adjusted to achieve the desired treatment efficiency at given operational costs. It is also important to note that there was an optimal aeration level. To consider this finding may limit costs at field-scale applications.

There are two potential limitations of the present study for translating its results to full-scale systems. First, the present investigation was carried out in batch mode. Several studies with FTW at scales ranging from laboratory to field scale have shown that results gained at smaller scales are essentially valid for the field scale, however, it is not a given that this is always the case. Secondly, long-term effects were not investigated in this study. The removal of hydrocarbons during the continuous operation of FTWs will have to be investigated in future work. Aspects of the FTW such as vitality of plants, dimensions of root network, i.e., a volume ratio of root network to free water, will change over time and may affect treatment performance.

### Cost-effectiveness ratio (CER)

In this study, CER was estimated yearly. At first, the total present value cost (pvc) for a single 1000 L wetland system was calculated as Eq. (4).

4 $${\text{pvc }}\left( {\$ {581}} \right) \, = {\text{ pvc}}_{{{\text{ic}}}} \left( {\$ {456}} \right) \, + {\text{ pvc}}_{{{\text{om}}}} \; \left( {\$ {125}} \right).$$

Then, CER_total_ for 12 months of operation was calculated by dividing pvc by the volume of water receiving treatment, multiplying the number of required treatments (n) (Eq. 5).

5 $$CER\, \left(total\right)=\frac{PV\, of\, total\, costs \left(\$581\right)}{ volume \,of\, water\, receiving\, treatment \; \left(12,000\, {\text{L}}\right)}\times \text{ n},$$

where n is a factor representing the number of times the system has been operated.

This indicated that, by following RSM, we can reduce the treatment cost up to $0.048n per liter of total wastewater receiving treatment.

RSM was successfully applied to optimize the abiotic variables nutrients concentrations, surfactant addition, aeration, and retention time for the attenuation of hydrocarbons from oil-contaminated water in a mesocosm-scale FTW experiment. The optimum values of the operational parameters were at a crude oil concentration of 0.758%, aeration 1.178 L of air min^−1^, a surfactant concentration of 0.006%; a nutrient ratio of 1.20; and retention time of 23.6 days for maximum hydrocarbons removal from the water, which resulted in a predicted and experimental attenuation of 98% and 95%, respectively. The performance of the system mainly depended on the retention time, but the initial oil concentration, surfactant concentration, nutrient ratio, and aeration rate also affected the removal of hydrocarbons from the water. Effect of salinity in crude oil wastewater treatment is nevertheless crucial, which may be included in the RSM design for future studies. Also, the results of RSM efficacy should be validated at pilot- and/or operational-scale for field-oriented conclusions. Thus, this study shows that the use of RSM is promising for reducing the costs of field-scale operation of FTW for hydrocarbon attenuation at oil processing sites.

## Materials and methods

### Chemicals and media

The crude oil was collected from an oil exploration and extraction company, Chakwal, Pakistan. All other chemicals used in the physicochemical and RSM studies were analytical grade and of the desired purity and were purchased from Merck, Germany, and Sigma-Aldrich, USA. A polystyrene sheet was purchased from Diamond Jumbolon Company and plastic tanks were purchased from the local market in Faisalabad.

### Generating the experimental design matrix with RSM

The combined effects of the five abiotic variables, inorganic nutrients (A), surfactant (B), aeration (C), hydrocarbon content (D), and retention time (E) were computed using RSM. Hydrocarbon and COD reduction, as well as plant growth, were analyzed as responses, with three levels each of the five variables as follows. Addition of inorganic nutrients (A): 140 mg L^−1^ and 14 mg L^−1^ nitrogen; and 19 mg L^−1^ and 1.9 mg L^−1^ phosphorus to make C:N:P ratio of 100:10:1 (level 1) and 100:1:1 (level 2), without added nutrients (level 3); concentration of sodium dodecyl sulphate as surfactant (B): 0.005% (w/v) and 0.01% (w/v) and 0% as level 1, 2 and 3, respectively; aeration (C): 1 L min^−1^, 2 L min^−1^, and zero as level 1, 2 and 3, respectively; hydrocarbon concentration (D): 0.5, 0.75 and 1% (w/v) as level 1, 2 and 3, respectively; and retention time (E): 8, 16 and 24 days. A CCD with the five variables at each of its three levels was generated by the Design Expert of RSM. The design matrix consisted of 32 factorial points, 10 axial points, and 8 central points, resulting in a total of 50 experimental runs (Table 1).

### Mesocosm setups and operation

The 50 experimental runs were established as triplicate mesocosm set ups (3 L) at the National Institute for Biotechnology and Genetic Engineering (NIBGE), Faisalabad, Pakistan. The experiment was set up at ambient temperature and light (April–May, 2021) at NIBGE, Faisalabad (31° 25′ 0″ N, 73° 5′ 28″ E), and the average day/night temperatures were 32 °C/18 °C. Per setup, three seedlings of common reed (*Phragmites australis*), each ~ 60 cm high and 45–65 g in weight were hydroponically grown in plastic pots with tap water for two months (Fig. 1). The characteristics of the tap water are shown in Table 4. Diammonium phosphate (500 mg) was added to each pot to support plant growth. The crude oil was collected from an oil drilling company and mixed in the water at different concentrations (0.5, 0.75, and 1%, w/v). A commercially available surfactant (Tween-20) was added to the water at three different levels (0. 0.005, and 0.01%, w/v). Air (0, 1, 2 L min^−1^) was provided in the water with the help of an electric pump. Floating rafts of appropriate dimensions were prepared using polyethylene-based roof insulation rolls (Jumbolon Rolls, manufactured by Diamond Foam Company, Pakistan), which are made of closed-cell polyethylene foam; for details: http://www.jumbolon.com/jumbolon-rolls^36^. The holes were made in the center of the raft and the seedlings were fixed in the holes with the help of coconut shaving and soil. Permissions or licenses were obtained to collect seedlings of *P. australis.* All the experiments were performed in accordance with relevant guidelines and regulations.

**Table 4:** The physicochemical parameters of water prior to the addition of crude oil.

Parameter	Value
pH	7.5
Total dissolved solids (mg L^−1^)	366
Electrical conductivity (mS cm^−1^)	0.57
Calcium (mg L^−1^)	32
Chlorides (mg L^−1^)	29
Magnesium (mg L^−1^)	18
Sodium (mg L^−1^)	26
Potassium (mg L^−1^)	11
Nitrogen	0
Phosphorus	0
*Escherichia coli* (cfu/100 mL)	0
Total coliform (cfu/100 mL)	0

*cfu* Colony forming unit.

### Analytical methods

The hydrocarbon fraction (mainly C10–C30 alkanes) in the water samples was determined as previously reported^37–39^. In brief, samples were extracted using *n*-hexane as a solvent, and the total hydrocarbon content in the extracts was determined with a Spectrum Two Environmental Hydrocarbon Analysis System (Perkin Elmer, USA). The solvent *n*-hexane was analyzed as a negative control. The chemical oxygen demand (COD) was measured with the standard method 5210B^40^.

Plant growth and biomass were determined at the end of the experiment. Shoots and roots were harvested above and below 2.5 cm of the floating raft, respectively. Their lengths were measured and their fresh and dry biomasses were determined using an analytical balance as described previously^41^.

### RSM model building

The results of the mesocosms experiment namely hydrocarbon reduction, COD removal, and plant growth were used for RSM modeling to get optimized values of each variable. A quadratic polynomial equation was used as a model to approximate the mathematical relationship of these five variables and their corresponding responses as presented in Eq. (6).

6 $${\text{Y}} = {\text{ a}}_{0} + {\text{ a}}_{{1}} {\text{A }} + {\text{ a}}_{{2}} {\text{B}} + {\text{ a}}_{{3}} {\text{C }} + {\text{ a}}_{{4}} {\text{D }} + {\text{a}}_{{5}} {\text{E }} + {\text{ a}}_{{{12}}} {\text{AB }} + {\text{ a}}_{{{13}}} {\text{AC }} + {\text{a}}_{{{14}}} {\text{AD}} + {\text{a}}_{{{15}}} {\text{AE}} + {\text{ a}}_{{{23}}} {\text{BC }} + {\text{ a}}_{{{24}}} {\text{BD }} + {\text{ a}}_{{{25}}} {\text{BE }} + {\text{a}}_{{{34}}} {\text{CD }} + {\text{a}}_{{{35}}} {\text{CE }} + {\text{ a}}_{{{45}}} {\text{DE }} + {\text{ a}}_{{{11}}} {\text{A2 }} + {\text{ a}}_{{{22}}} {\text{B2 }} + {\text{ a}}_{{{33}}} {\text{C2 }} + {\text{a}}_{{{44}}} {\text{D2}} + {\text{a}}_{{{44}}} {\text{E2}},$$

where Y is the predicted response value, a_0_ is the value of the fitted response at the center point of the design; a_1_, a_2_, a_3_, a_4_ and a_5_ are the linear coefficients; a_12_, a_13_, a_23_ … are the cross product coefficients; a_11_, a_22_, a_33_, a_44_, and a_55_ are the quadratic coefficients. The design matrix with five variables and the three coded levels (− 1, 0, + 1) is presented in Table 1. All the variables were taken at the coded values. F test and computation of *R*^2^ (correlative coefficient value) were carried out to check the statistical significance and quality fit of the mathematical model, respectively.

### Cost-effectiveness ratio

To further assess the utility of RSM in terms of parameters optimization, we calculated the cost-effectiveness ratio (CER) for a FTW system having a single optimized treatment instead of a multiple remediation setup^33^. For this study, our CER results are based on a pilot-scale FTW that has been used in our earlier studies (e.g.^41–43^). Because more than one variable is tested in each study, the cost may increase based on the number of variables and responses in a randomized complete block design (RCBD), i.e., 5 variables and 3 responses in this study. Hence, to have a single system operating under the best conditions, total costs could be reduced significantly.

For a single FTW treatment system, the total cost is usually divided into capital and operational/maintenance costs. The capital costs include pollution investigation, preparation of the wetland architectural design, and purchase of material such as plants, rafts, and pumps. Operational/maintenance costs included labor costs, routine investigations, pump operation, and overall maintenance. The total present value cost (pvc) for a FTW system is calculated by Eq. (7).

7 $${\text{pvc }} = \, \left( {{\text{pvc}}_{{{\text{ic}}}} + {\text{ pvc}}_{{{\text{om}}}} } \right),$$

where pvc_ic_ is the present value of wetland capital cost, pvc_om_ is the present value of operational and maintenance cost, and n is a factor representing number of times the system has been operated.

The operational cost is calculated for 1 year, therefore, results of CER are estimated on a yearly year basis, which has been calculated by dividing pvc by the volume of water receiving treatment, multiplying the number of required treatments (n) (Eq. 8).

8 $$CER \; \left(total\right)=\frac{PV\, of\, total \,costs }{ volume \,of\, water\, receiving\, treatment \left(12,000 {\text{L}}\right)}\times \text{ n}.$$

### Statistical analysis

The quadratic models were fitted using RSM for three responses (hydrocarbons reduction, COD reduction, and plant biomass), which described the mathematical relationship between each term in the model and response. Here, analysis of variance (ANOVA) was used to split the total variation into different model components; whereas, to check the significance of each component, F-test was used. F-Test is the ratio of two mean squares (specific component divided by the error term). Lastly, to decide the significance of each term, a comparison was made between two mean squares (as shown in Table 3). The significance of each term was assessed by calculating the p-values against the F-Test value of each term to decide whether the model term contributes significantly to the response variable.

The mesocosms parameters were also subjected to ANOVA using Statistix 9. The post-hoc Tukey’s HSD test was applied for multiple comparisons and p-values were considered to be significant at p < 0.05.
